# Supplementary material for: Deletion of wfs1 Impairs Oligodendrocyte Precursor Cells Dorsal Distribution and Myelination Through the wfs1-hmgcs1 Axis in Zebrafish
Source: Biology (Basel). 2026 Aug 16;15(16):1402. doi: 10.3390/biology15161402 (PMC13510151; doi:10.3390/biology15161402)
Supplement: Supplementary file 1 [file biology-15-01402-s001.zip › File S2-The original Western blot images.pdf]

Supplementary Materials

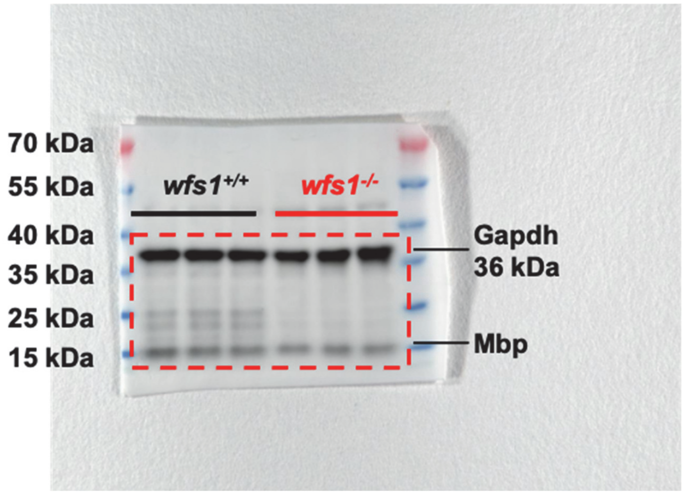

Figure S1. Western blot membrane of Mbp (17~25 kDa) protein detected with rabbit anti-Mbp (1:1000; Boster, China) antibody. Zebrafish larvae at 5 dpf were homogenized in RIPA lysis buffer supplemented with protease inhibitors, and protein concentration was determined by BCA assay. Proteins were separated by 10% SDS-PAGE and transferred onto PVDF membranes (Millipore, USA) pre-activated in methanol. Membranes were blocked with 5% skim milk in TBST for 1 hour at room temperature, followed by overnight incubation with primary antibody at 4°C. After washing with TBST, membranes were incubated with HRP-conjugated goat anti-rabbit secondary antibody (1:5000; Proteintech, USA) for 1 hour at room temperature. Protein signals were detected using ECL chemiluminescence substrate (Thermo Fisher, USA) and captured with a chemiluminescence imaging system. Band intensities were quantified using ImageJ software (version 1.53q) and normalized to Gapdh. Lanes 1-3: *wfs1*$^{+/+}$; lanes 4-6: *wfs1*$^{-/-}$.

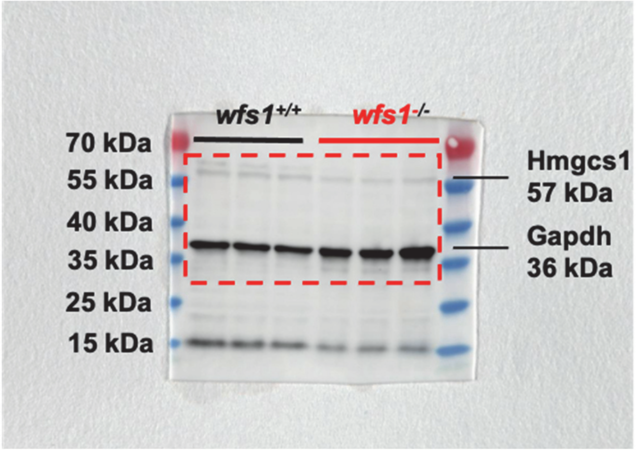

Figure S2. Western blot membrane of Hmgcs1 (~57 kDa) protein at 4 dpf detected with

rabbit anti-Hmgcs1 (1:1000; Proteintech, USA) antibody. Zebrafish larvae at 4 dpf were homogenized in RIPA lysis buffer supplemented with protease inhibitors, and protein concentration was determined by BCA assay. Proteins were separated by 10% SDS-PAGE and transferred onto PVDF membranes (Millipore, USA) pre-activated in methanol. Membranes were blocked with 5% skim milk in TBST for 1 hour at room temperature, followed by overnight incubation with primary antibody at 4°C. After washing with TBST, membranes were incubated with HRP-conjugated goat anti-rabbit secondary antibody (1:5000; Proteintech, USA) for 1 hour at room temperature. Protein signals were detected using ECL chemiluminescence substrate (Thermo Fisher, USA) and captured with a chemiluminescence imaging system. Band intensities were quantified using ImageJ software (version 1.53q) and normalized to Gapdh. Lanes 1-3: *wfs1*$^{+/+}$; lanes 4-6: *wfs1*$^{-/-}$.

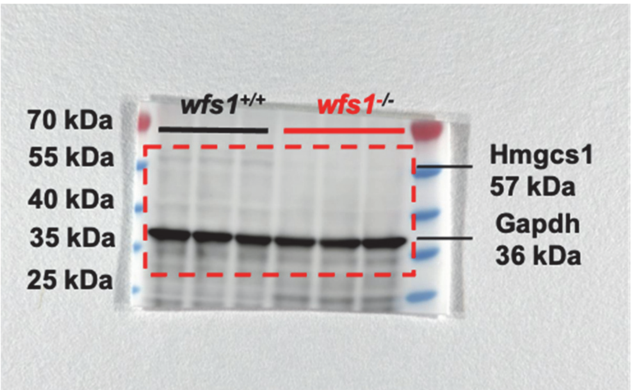

Figure S3. Western blot membrane of Hmgcs1 (~57 kDa) protein at 5 dpf detected with rabbit anti-Hmgcs1 (1:1000; Proteintech, USA) antibody. Zebrafish larvae at 5 dpf were homogenized in RIPA lysis buffer supplemented with protease inhibitors, and protein concentration was determined by BCA assay. Proteins were separated by 10% SDS-PAGE and transferred onto PVDF membranes (Millipore, USA) pre-activated in methanol. Membranes were blocked with 5% skim milk in TBST for 1 hour at room temperature, followed by overnight incubation with primary antibody at 4°C. After washing with TBST, membranes were incubated with HRP-conjugated goat anti-rabbit secondary antibody (1:5000; Proteintech, USA) for 1 hour at room temperature. Protein signals were detected using ECL chemiluminescence substrate (Thermo Fisher, USA) and captured with a chemiluminescence imaging system. Band intensities were quantified using ImageJ software (version 1.53q) and normalized to Gapdh. Lanes 1-3: *wfs1*$^{+/+}$; lanes 4-6: *wfs1*$^{-/-}$.
